# Mixing Carrots and Sticks to Conserve Forests in the Brazilian Amazon: A Spatial Probabilistic Modeling Approach

**DOI:** 10.1371/journal.pone.0116846

**Published:** 2015-02-04

**Authors:** Jan Börner, Eduardo Marinho, Sven Wunder

**Affiliations:** 1 Center for Development Research (ZEF), University of Bonn, Walter-Flex-Str. 3, 53113 Bonn, Germany; 2 Center for International Forestry Research (CIFOR), Rua do Russel 450/ s.601, 22.210-010 Rio de Janeiro-RJ, Brazil; University of Waterloo, CANADA

## Abstract

Annual forest loss in the Brazilian Amazon had in 2012 declined to less than 5,000 sqkm, from over 27,000 in 2004. Mounting empirical evidence suggests that changes in Brazilian law enforcement strategy and the related governance system may account for a large share of the overall success in curbing deforestation rates. At the same time, Brazil is experimenting with alternative approaches to compensate farmers for conservation actions through economic incentives, such as payments for environmental services, at various administrative levels. We develop a spatially explicit simulation model for deforestation decisions in response to policy incentives and disincentives. The model builds on elements of optimal enforcement theory and introduces the notion of imperfect payment contract enforcement in the context of avoided deforestation. We implement the simulations using official deforestation statistics and data collected from field-based forest law enforcement operations in the Amazon region. We show that a large-scale integration of payments with the existing regulatory enforcement strategy involves a tradeoff between the cost-effectiveness of forest conservation and landholder incomes. Introducing payments as a complementary policy measure increases policy implementation cost, reduces income losses for those hit hardest by law enforcement, and can provide additional income to some land users. The magnitude of the tradeoff varies in space, depending on deforestation patterns, conservation opportunity and enforcement costs. Enforcement effectiveness becomes a key determinant of efficiency in the overall policy mix.

## Introduction

While globally, tropical forest cover loss may have increased over the past decade [1], in Brazil annual forest conversion has dropped substantially. According to the Brazilian Space Research Center (INPE), Amazon deforestation in 2012 was 13,750 km^2^ lower than the historical average and roughly 700 km^2^ below the ambitious national policy target for the 2011–2015 period [2]. Evaluation studies suggest that changes in the enforcement strategy of the Brazilian Forest Law (*Código Florestal*) have significantly contributed to reducing deforestation in Brazil’s Amazon region [3, 4].

Despite the success in reducing annual primary forest loss, non-compliance with the forest retention standard of the Forest Law is widespread due to high historical deforestation rates. In response to political pressure from a rural development lobby that has faced increasing opportunity costs from what it sees as excessively strict environmental regulations, the federal government has revised the law in 2012. The reformed law reduces the liabilities of past offenders, requires restoration of illegally deforested land, and also broadens the scope for legal forest conversion [5].

From an environmental policy perspective, less ambitious conservation standards provide scope for managing land use and land cover change through combinations of command-and-control (C&C) measures and voluntary conservation incentives, such as payments for environmental services (PES). This is because voluntary conservation incentives are challenging to design in a context where deforestation is predominantly illegal [6]. An increasing use of positive incentives could eventually help making the dramatic reduction in deforestation politically sustainable, by counteracting imminent pressures to revert the achieved restrictions in land use expansion [7].

Against the backdrop of successful conservation law enforcement and the revision of the national Forest Law, the environmental policy debate is indeed increasingly embracing incentive-based approaches to forest conservation. Various federal states, such as Acre, Amazonas, Sao Paulo, and Rio de Janeiro, have adopted legislation to facilitate the use of public funds for PES-type of arrangements, and at the national level a bill to create a national PES program (PL 792/2007) has already passed several administrative hurdles.

This paper focuses on the challenge of designing a policy mix that integrates C&C enforcement policies with PES-based instruments. The notion that multiple policy objectives cannot be achieved by a single policy instrument goes back to the economist Tinbergen [8]. According to Lehmann [9], policy mixes can also perform better than single instruments when market failures are caused by multiple reinforcing factors, such as pollution externalities, technology spillovers, and asymmetric information. In the context of conservation, adding a policy instrument by mixing regulatory disincentives with incentive-based instruments comes with the potential to simultaneously improve the provision of environmental public goods and the income of land users [10]. The evidence-base on the effectiveness of policy mixes in the conservation sector is still thin, however, including because evaluation studies have so far focused primarily on single instrument assessments [11–13]. Quantitative tools and concepts for the analysis and evaluation of conservation policy mixes have been developed only recently [13, 14].

While cost-neutral from a social welfare perspective, the implementation of large-scale government-led PES schemes in the Brazilian Amazon would come with a significant additional financial burden for the public administration. Previous work indicates that the implementation costs of forest law enforcement in the Brazilian Amazon are comparatively low [15], compared with estimates of incentive-based approaches to reducing deforestation [6, 16]. In fact, forest law enforcement could be a net source of government revenue if offenders were successfully pursued to pay their fines. Currently, however, legal coercion takes place primarily through costly *in situ* confiscation and cross-compliance measures, such as credit access restrictions [17].

Beyond implementation costs, PES and C&C have dramatically different economic implications for land users. While C&C reflects the “polluter pays” principle (land users bear compliance costs), a PES-based approach adopts a “beneficiary pays” paradigm, i.e., land users are fully or partially compensated for the opportunity costs of conservation. Moreover, equity implications arise, depending on how property rights and opportunity costs are distributed in space. For example, high concentration in (forest-) land ownership in the Brazilian Amazon implies that, absent any social targeting efforts, large landholders would benefit most from PES [18].

Our main intended contribution to the literature on conservation policies here is a spatially explicit model to simulate conservation efficiency, income, and equity effects of alternative C&C with PES policy mixes. The modelling approach is new in that it (1) explicitly accounts for the costs of implementing alternative policy mixes, and (2) integrates bottom-up conservation opportunity cost estimates with remotely sensed deforestation data to estimate continuous avoided deforestation cost curves at sub-district spatial scales. We use our model to address two research questions:

1. At what implementation costs could PES be integrated in the existing C&C framework to achieve different conservation targets?
2. What tradeoffs are associated with alternative combinations of PES and C&C, in terms of implementation costs and landholder incomes?

In the following section, we outline the conceptual framework that motivates land use and enforcement decisions in our simulation model (details are given in S1 Appendix). Section 3 documents data sources and model implementation strategies (for details, see S2 Appendix). Section 4 presents our main results. We discuss benefits and caveats of the modelling approach in Section 5 and conclude with policy implications in Section 6.

### Conceptual framework

Conservation policies and their effect on deforestation have been analyzed before, for example, using general and partial equilibrium models, or rule-base spatial simulation models [5, 19]. The approach we use here aims to combine advantages of both modeling strategies. As in general or partial equilibrium models, we model deforestation decisions as economically motivated. However, by disaggregating bottom-up opportunity cost estimates [20], we can analyze the spatial distribution of policy performance as in rule-based spatial simulation models. In addition, by explicitly modeling the environmental enforcement strategy as a constrained optimization problem, we introduce a notion of imperfect policy performance, thus relaxing the controversial assumption of perfect compliance that is frequently made in environmental policy assessments [21].

We start out from Becker’s [22] standard model of enforcement, where the private agent’s decision to engage in a profitable illegal activity (here, deforestation) depends on the expected return and the cost of punishment. Faced with the decision to deforest or not, our representative land user’s decision problem is:

$$\max_{d}f_{\left(d\right)}$$ (Eq. 1a)

$$\max_{d'}f_{\left(d'\right)}-d'pF+\mathrm{PES}\left(d-d'p\right)\;\mathrm{with}\;d'<d$$ (Eq. 1b)

Where *d* and *d’* are the levels of deforestation (in hectares) before (baseline deforestation) and after the policy mix implementation, *f(.)* represents per hectare profits of deforestation with decreasing returns to *d* and *d’*. Decreasing returns to deforestation imply that, in each grid cell, the marginal return to forest clearing decreases with each additional plot cleared as in a classical land rent model [23], for example, due to increasing costs of access to remote production sites. *p* is the probability to receive a fine (*F*) or any other coercive measure of similar value per hectare (e.g., confiscation of assets).

Eq. 1a represents the land user’s decision problem in the absence of policy incentives. The land user maximizes the total return to deforestation by changing the amount of forest cleared (*d*). Eq. 1b depicts deforestation decisions with policy incentives. We here go beyond the standard enforcement model by adding a PES component, and thus creating a policy mix. *PES* is a per hectare one-off payment conditional on avoided deforestation (vis-à-vis the baseline), where the conditionality is enforced with the same probability as the fine. We thus assume that the incentive component of the policy mix (*PES*) is enforced through the same mechanism (i.e. field inspections) that is used by the Brazilian federal government to enforce conservation regulations.

Hence, land users receive the full per hectare payment only for the avoided share of baseline deforestation, whereas the amount received for residual deforestation depends on the size of *p* (third term in Eq. 1b). If *p < 1*, a share of PES, namely *1 - p*, will be ineffective as a conservation incentive and accrue to the land user as an imperfect enforcement rent.

Note that the payment can be made jointly conditional on legal compliance and voluntary conservation actions that exceed legal standards to avoid that land users are being compensated merely to comply with the law, i.e. moral hazard. This is a common practice in national PES schemes, such as in the Costa Rican national PES scheme [24], and even in the *Bolsa Floresta* PES program implemented in Amazonas state [25]. Our model does not include the possibility of moral hazard, which is why we do not account for the extra costs of such legally additional conservation actions.

Setting the first derivative of Eq. 1b equal to zero, the optimal level of illegal deforestation is given by:

f'(d*)=p(F+PES) (Eq. 2)

That is, risk-neutral land users will deforest up to the point where the marginal return to deforestation is equal to the expected policy incentive. In the absence of incentives, the optimal deforestation level corresponds to the point where the marginal return to deforestation equals zero.

Inspired by Albers [26] and following [27], we model the environmental protection agency (EPA) as an independent decision-maker that seeks to minimize illegal deforestation subject to a fixed budget constraint. In contrast with Albers’ protected area model, we assume that patrolling as a preemptive enforcement strategy is not an option in extensive tropical forest landscapes like the Amazon region. Instead the EPA establishes enforcement pressure *ex-post*, by inspecting remotely sensed deforestation patches and holding land users responsible if liability can be established. Deterrence is thus assumed to be achieved by demonstrating visibly to both punished and non-punished land users in the targeted region that illegal deforestation is not without economic consequences. In practice, *ex post* liability establishment often hinges on the nature of property rights.

Field inspections are costly and due to the budget constraint the EPA cannot inspect all deforestation patches in a given year. As a result, the probability of enforcement (*p* in Eqs. 1b and 2) will be smaller than one in many areas. The EPA’s decision problem in a given year can thus be conceived as follows:

$$\begin{array}{c} \max_{p}\sum_{i=1}^{I}d{'}_{i}p_{i}\\ s.t.\sum_{i=1}^{I}p_{i}TC_{i}+nd_{i}TC_{i}^{'}\leq B \end{array}$$ Eq. 3

That is, the EPA seeks to minimize illegal deforestation by maximizing area of inspected illegal deforestation. In Eq. 3, *I* represents the target area that consists of spatial units *i. d’* is the level of deforestation after the policy mix and *TC* is the travel cost of inspecting one deforestation patch in *i, TC’* is the average cost of visiting additional patches in *i, nd* is the number of patches in *i*, and *B* is the budget of the EPA. Based on this decision framework, we would expect enforcement probabilities to be high in spatial units with high deforestation levels and low inspection costs and *vice versa*. In S1 Appendix (Figure A1) we show empirically that the enforcement strategy of the Brazilian EPA (IBAMA) does indeed exhibit this pattern.

If the EPA allocates enforcement missions according to Eq. 3 over a period of several years (with natural variation in *d* over time), land users in each spatial unit may or may not be inspected in a given year depending on the spatial pattern of deforestation in that year. Assuming static expectations the land users’ expected enforcement probability *p* in spatial unit *i* is then given by the historical frequency of inspections, i.e. $p_{i}=\frac{\mathrm{years with inspections}}{\mathrm{total number of years with enforcement}}$. We exploit this notion when we generate spatially explicit versions of policy mix scenarios. To spatialize the enforcement strategy that results from the optimization in Eq. 3, we assume that the enforcement probability (*p*_i_) is a logistic function of inspection costs and deforestation levels.

**Fig 1:**
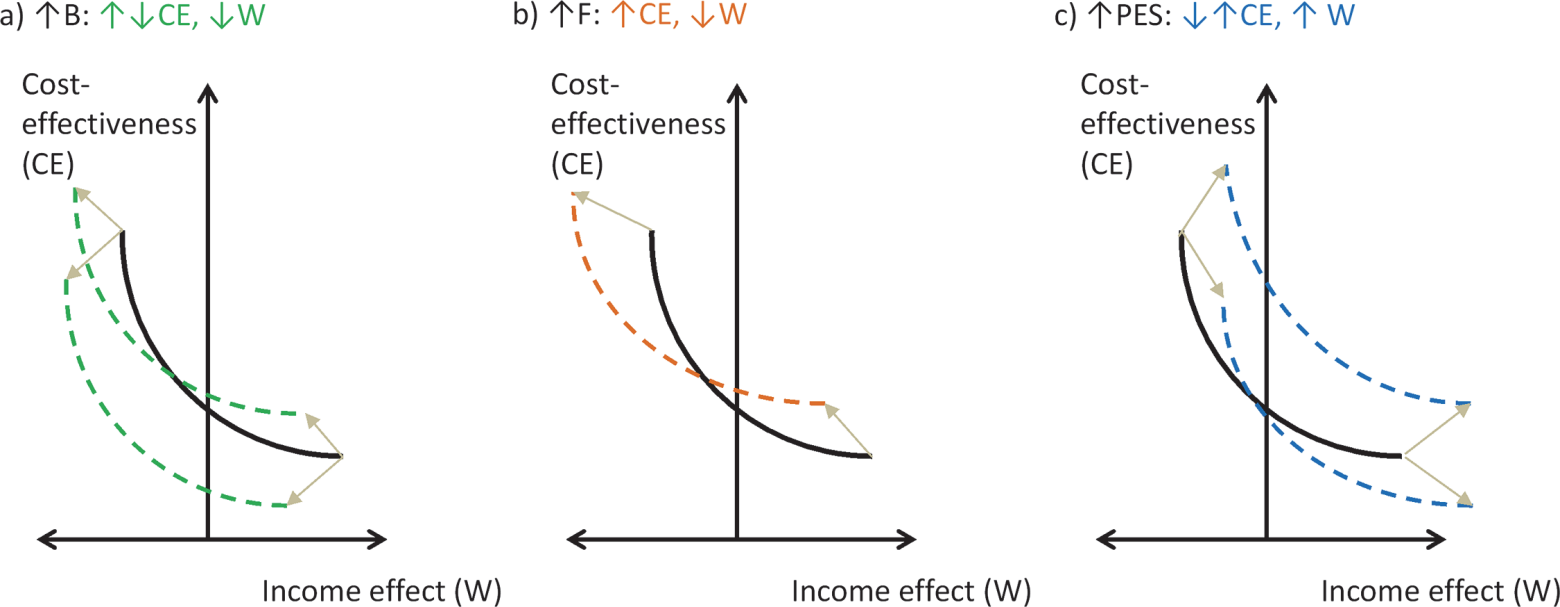
Expected effects of changing policy mix parameters on key policy performance indicators. B, F, and PES are policy design parameters. CE and W are policy targets or outcome variables. Black arrows depict directions of change in policy design parameters and colored arrows depict expected effects.

Based on this framework, the government to which the EPA reports can influence forest law enforcement through three levers: the EPA’s budget (*B*), the size of the per hectare disincentive (*F*), and the size of the per hectare incentive (PES). A policy mix is defined as a combination of *B, PES*, and *F*. Fig. 1 depicts the potential outcomes of alternative decisions on these policy design parameters in terms of land users’ income and conservation cost-effectiveness. Along the curves in all panels of Fig. 1, the level of avoided deforestation is constant for alternative combinations of *F* and *PES*.

Consider panel a): by increasing the EPA’s budget (B), the government will increase the agency’s mobility and thus inspection frequency in the target area. As a result, land users will face higher enforcement probabilities. Whether the policies’ cost-effectiveness (CE) increases depends on two factors, namely the nature of coercive measures and current compliance levels. If forest law is enforced through fines, higher enforcement probabilities can result in higher fine revenues and thus make the policy more cost-effective. Yet, if forest law is primarily enforced through *in situ* measures that generate little or no revenues, such as asset confiscation, the policy’s implementation costs will increase and the effect of a budget increase will depend on compliance levels. At low compliance levels, increases in enforcement pressure are likely to boost the policy’s effectiveness. At high compliance levels, however, increases in enforcement costs can outperform gains in effectiveness. Increases in *B* will always have negative income effects by way of increasing the enforcement probability.

Now turn to panel b): here increasing the per hectare disincentive (F) tends to increase both the policies effectiveness and fine revenues and thus results in higher cost-effectiveness. But land users will pay higher fines, and thus be worse off in terms of income.

Increasing the per-hectare incentive (PES), as in panel c), will always increase policy implementation costs, but overall effects on cost-effectiveness depends on whether gains in conservation effectiveness are larger than increases in these costs. Income effects for land users tend to be positive.

As a result, the government will often face a tradeoff between cost-effectiveness and income effects of alternative mixes of disincentive and incentives for conservation: the larger the relative emphasis on PES, the less cost-effective is the policy, but the more it mitigates land user’s private opportunity costs.

## Data & Methods

### a) Study area and data sources

**Fig 2:**
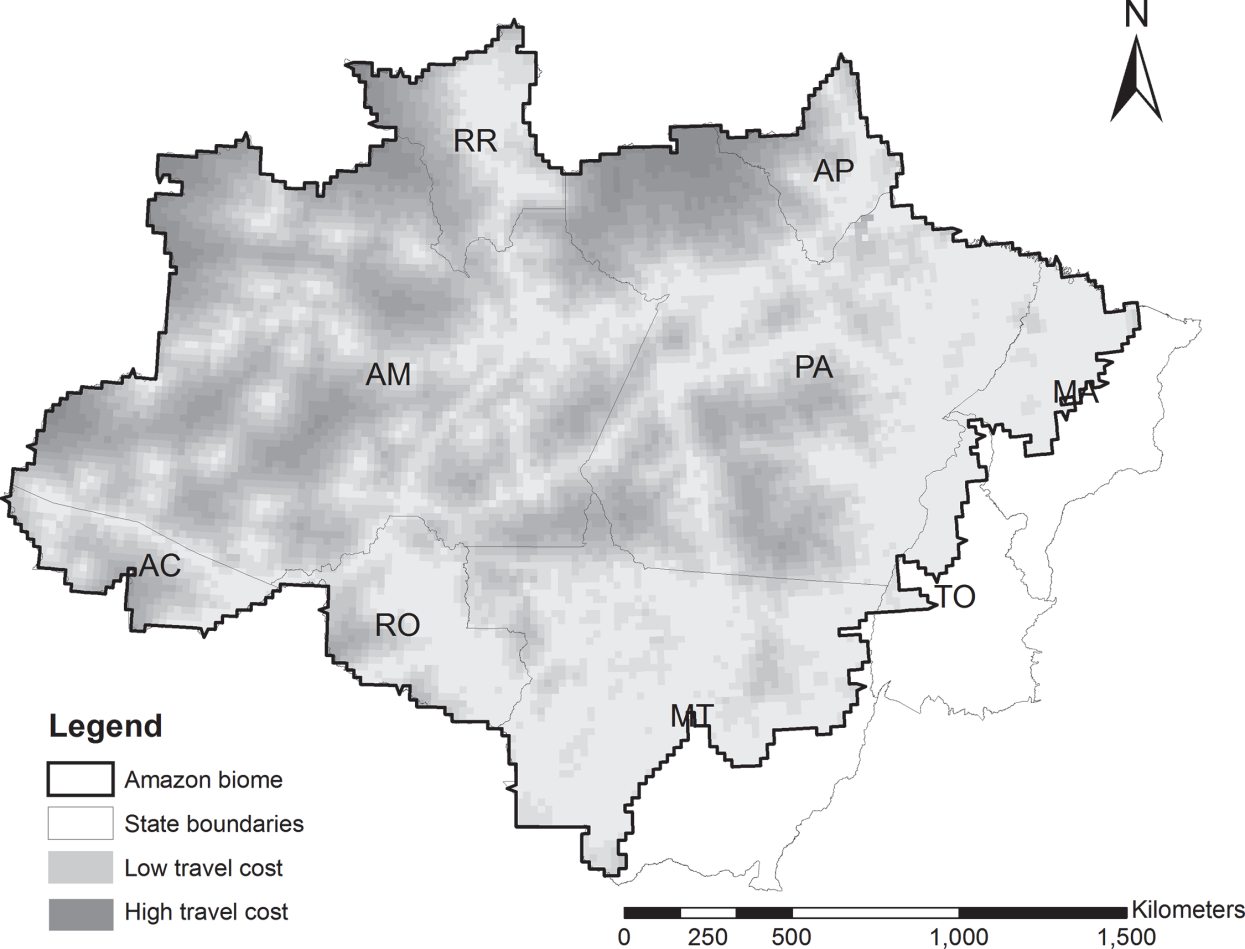
Study area and variation in inspection costs.

We implement the model outlined in the previous section in the context of the Brazilian Amazon (Fig. 2). Our analysis is based on the data sources in Table 1. All analyses are done at the scale of 20×20 km grid cells.

**Table 1:** Data sources.

**Data type**	**Source**
1. Remotely sensed deforestation data	INPE-PRODES at (accessed in 2014): http://www.obt.inpe.br/prodes
2. Municipal-level average per hectare profit of deforestation (i.e., conservation opportunity costs)	Börner et al. (2010)
3. Travel (including inspection) costs from district centers to grid cell centroids	Börner et al. (2014)

### b) Opportunity cost estimation

A key parameter in our analysis is the opportunity cost of forest conservation *f(.)* introduced above in Eq. 1. In this paper, we move beyond the approach in [27] by introducing the notion of diminishing economic returns to deforestation (see details in S2 Appendix). We interpret the estimated municipal-level average per hectare profits (see Table 1) as the weighted average of profits obtained from deforestation in a given district. Variations in deforestation across the 20×20 km grid cells in a district should then reflect local variations in opportunity costs. That is, if no deforestation occurs, it is because expected profits, at least for the time being, are negative (e.g. because of marginal returns from the land and/or excessive costs of mobilizing the additionally needed capital or labor). Conversely, if deforestation occurs, it is because expected profits are positive. Within a grid cell, we thus assume that the spatial distribution of profits follows a normal distribution, with likely both positive and negative values. As shown in S2 Appendix, the parameters for the distribution of profits can be deduced from a grid cell’s observed deforestation rate. For a given district, the parameters of the normal distribution in each of the grid cells are estimated such that the deforestation-area weighted average of their positive ranges corresponds to the district-level average profit. Modelling opportunity costs as documented in S2 Appendix results in opportunity costs that are negatively correlated with field-based law enforcement costs (*r* = −0.33).

### c) Simulating policy mixes

Estimating sub-district level opportunity costs, as outlined above, results in individual profit functions for each of the grid cells in our study area. Profits exhibit diminishing returns to the scale of deforestation, or increasing marginal opportunity costs of avoided deforestation. The effect of policy-induced incentives (PES) and disincentives (F) on deforestation can be simulated by shifting the underlying normal distributions to the right or left, respectively. For our study area we can then calculate the cost-effectiveness of the policy mix as follows:

$$CE=\frac{\sum_{i=1}^{I}\Delta d_{i}}{B+\sum_{i=1}^{I}\mathrm{PES}\;(d_{i}-d_{i}^{'}p)}$$ (Eq. 4)

The numerator of Eq. 4 is the total amount of avoided deforestation for a given policy mix. The implementation cost of the policy (denominator of Eq. 4) is composed of the EPA’s budget *B*, the sum of all payments made to compliant land users over all grid cells *I* minus the sum of transfers recovered from non-compliant land users depending on the probability of enforcement (see previous section). *d’* is the level of deforestation after the policy mix was implemented and is determined by Eq. 1b. If, for simplicity, we assume here that *F* in Eq. 1b is a fine collected by the EPA then we can subtract $\left(\sum_{i=1}^{I}p_{i}Fd_{i}^{'}\right)$, i.e. the amount of fines successfully collected from non-compliant land users, from the denominator. *CE* thus represents the amount of avoided deforestation per monetary unit of implementation cost. Note that modelling policy implementation costs as in Eq. 4, we assume that the enforcement agency’s budget is independent from the budget used to pay land users for avoided deforestation [28]. While this reflects the current institutional set-up in the Brazilian environmental governance system, the policy mix could alternatively also be modelled as an optimal budget allocation decision. This would, however, require assumptions about policy-makers’ preferences regarding tradeoffs between conservation cost-effectiveness and income effects of the policy mix. Since our focus is on assessing the dimensions of this tradeoff we do not make such assumptions in this paper.

For each policy mix (B, PES, F) we can further calculate the aggregate income effect in the study area as:

$$W=\sum_{i=1}^{I}\mathrm{PES}(d_{i}-d_{i}^{'}p_{i})\;-p_{i}Fd_{i}^{'}-\Delta f_{i\left(.\right)}$$ (Eq. 5)

with $\Delta f_{i\left(.\right)}=f_{i\left(d\right)}-f_{i\left(d'\right)}$

In each 20×20 km grid cell *i*, the change in land-user income from the applied policy mix *vis-à-vis* the business-as-usual scenario (*laissez faire*) is composed by the three terms in Eq. 5: (1) the PES transfers to compliant land users and to non-compliant land users who were not held liable due to imperfect enforcement (*p < 1*), (2) the amount of fines *F* paid by non-compliant land users who were hold liable, and (3) the opportunity costs of compliant land users. In principle, PES and F can vary across grid cells according to social or environmental targeting criteria [29]. Alternative PES design options for the Amazon have been discussed in [6]. We thus limit ourselves here to the case of uniform per hectare payments in order to focus on the implications of mixing incentives with disincentives.

### d) Parameter choices

We simulate alternative policy mixes by varying one or more of the following input parameters: (1) EPA’s budged (*B)*, the per hectare disincentive of the C&C policy component (*F)*, and the per hectare incentive of the PES policy component (*PES)*. Table 2 documents the parameter ranges used in all simulations.

**Table 2:** Ranges of parameter values used in simulations.

**Parameter**	**Value range/estimation**	**Comments**
*d*(deforestation in base year)	Fixed for each grid cell based on observed deforestation in the years 2002–2004 prior to policy change in Brazil (see Table 1)	See methodological description in S2 Appendix
*TC* (inspection cost of visiting first deforestation patch in *i*)	Fixed for each grid cell estimated based on cost data from IBAMA and travel time map as documented in Börner et al. (2014)	
*TC’* (within grid cell inspection costs)	Fixed for each grid cell as average cost of travelling through half of the cell	
*nd* (number of additional deforestation patches per grid cell)	Estimated as a linear function of total deforestation per grid cell from historical deforestation data (0.035 patches per hectare)	
*B* (enforcement budget)	0 to BRL 50,000,000 - BRL 1 ∼ USD 0,45 (July 2014: http://www.oanda.com)	IBAMA’s 2004 operational budget for “environmental protection” at the national level was BRL 62 million. According to interviews with the agency, most of this budget is allocated to the Amazon region.
*F* (per hectare disincentive)	BRL 0–5000	BRL 5000 per hectare was the value of a fine for violating the forest retention standard according to the 1998 version of Law 9.605
*PES*(per hectare incentive)	BRL 0–5000	No established value yet at national level

## Results

We first look at the patterns of conservation opportunity costs that emerge from our approach to disaggregating conservation opportunity costs from district to grid cell scale. We then turn to policy scenario analyses with a focus on cost-effectiveness and income effects. All scenarios are generated by changes in the three key policy design parameters (*B, F, PES*), as outlined in Sections 2 and 3.

### a) The cost of avoiding deforestation

**Fig 3:**
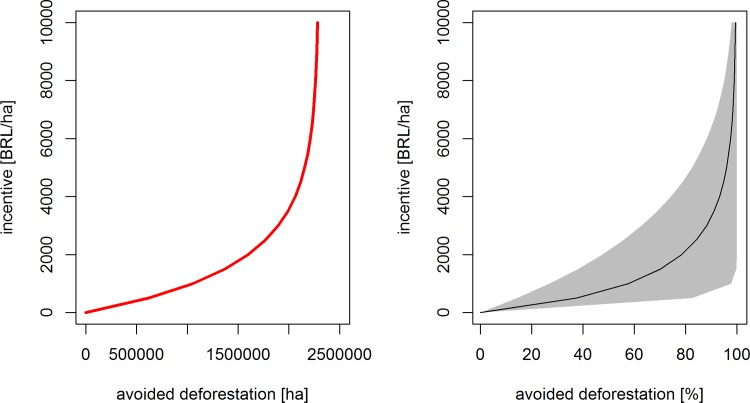
Avoided deforestation cost curve in low enforcement scenario (year 2004, baseline deforestation). Whole study area (left) and 0.05 to 0.95 percentile range across grid cells with deforestation.

The left panel in Fig. 3 depicts the avoided deforestation cost curve for the Amazon biome (see Fig. 2). The values on the vertical axis represent conservation incentives that are effectively delivered on the ground. In other words, based on cost considerations alone—and thus, abstracting from other preconditions for PES, such as clear land tenure [30] - half of the deforestation in the study area could be avoided by providing farmers with fixed per hectare compensation of slightly less than 1500 Brazilian *Reais* (BRL; see current conversion rate in Table 2). Note that the resulting vertical asymptote at approximately 2.2 million hectares corresponds to the observed decrease in deforestation between 2004 and 2012 (2.32 million hectares).

The right panel in Fig. 3 shows the variation in opportunity costs of avoiding deforestation across grid cells (shaded in grey). For example, the cost of avoiding half of the average pre-2005 deforestation level can vary between close to zero to almost BRL 2000 per hectare depending on where in the region a given grid cell is located.

### b) Efficiency-welfare tradeoffs

**Fig 4:**
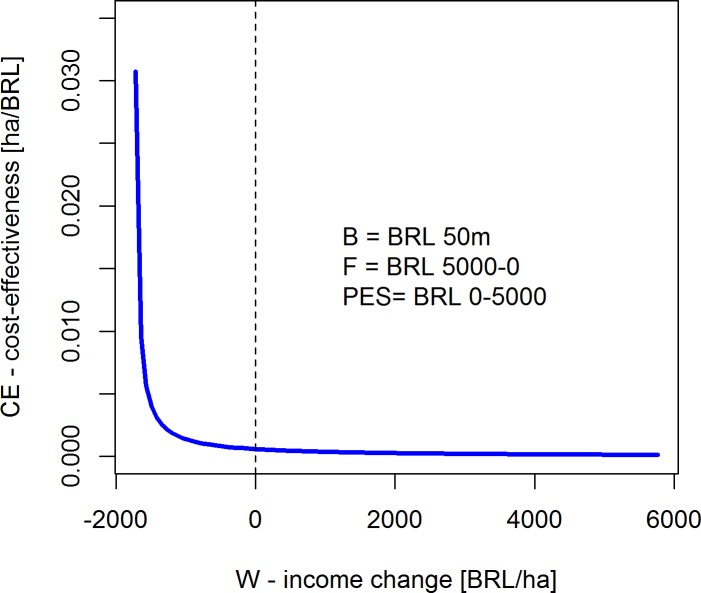
Cost-effectiveness (CE) versus income loss (W) tradeoff of alternative per hectare PES and F incentive mixes. Legend: Parameter values/ranges used in the simulation.

We hypothesized above that policy makers who intend to control Amazon deforestation through a mix of C&C disincentives and PES incentives will face a tradeoff between conservation efficiency and income losses by Amazon land users. Fig. 4 illustrates this tradeoff holding the enforcement budget (B) and the amount of avoided deforestation constant, while progressively moving from pure C&C to pure PES (from the left to the right) by gradually replacing *F* with *PES*. Fine revenues are set to zero reflecting the current reality of low fine collection in the Brazilian Amazon, i.e. the disincentive component of the policy is delivered through alternative coercive measures that generate no revenues to the EPA (see previous section).

We find that a purely C&C dominated policy mix achieves the highest cost-effectiveness with over 30 hectares of forest conserved per 1000 BRL invested in this policy. Gradually increasing the incentive component of the policy mix (PES), while reducing the disincentive component (F) by an equivalent amount, reduces cost-effectiveness. The sharp initial drop levels off quickly as PES sets in, and then asymptotically approaches zero. Average per hectare change in income is zero at fine levels (F) of approx. BRL 3500 and PES of approx. BRL 1500 per hectare. At this point, the cost-effectiveness of the policy mix is roughly 0.5 ha/1000 BRL, or 98.5% lower than in the pure C&C scenario.

Hence, making conservation in the Brazilian Amazon region income neutral will, at least on average, require a greatly more expensive policy mix (> BRL 2.5 billion per year) from a budgetary perspective.

### c) Spatial patterns of policy implementation costs and fine revenues

**Fig 5:**
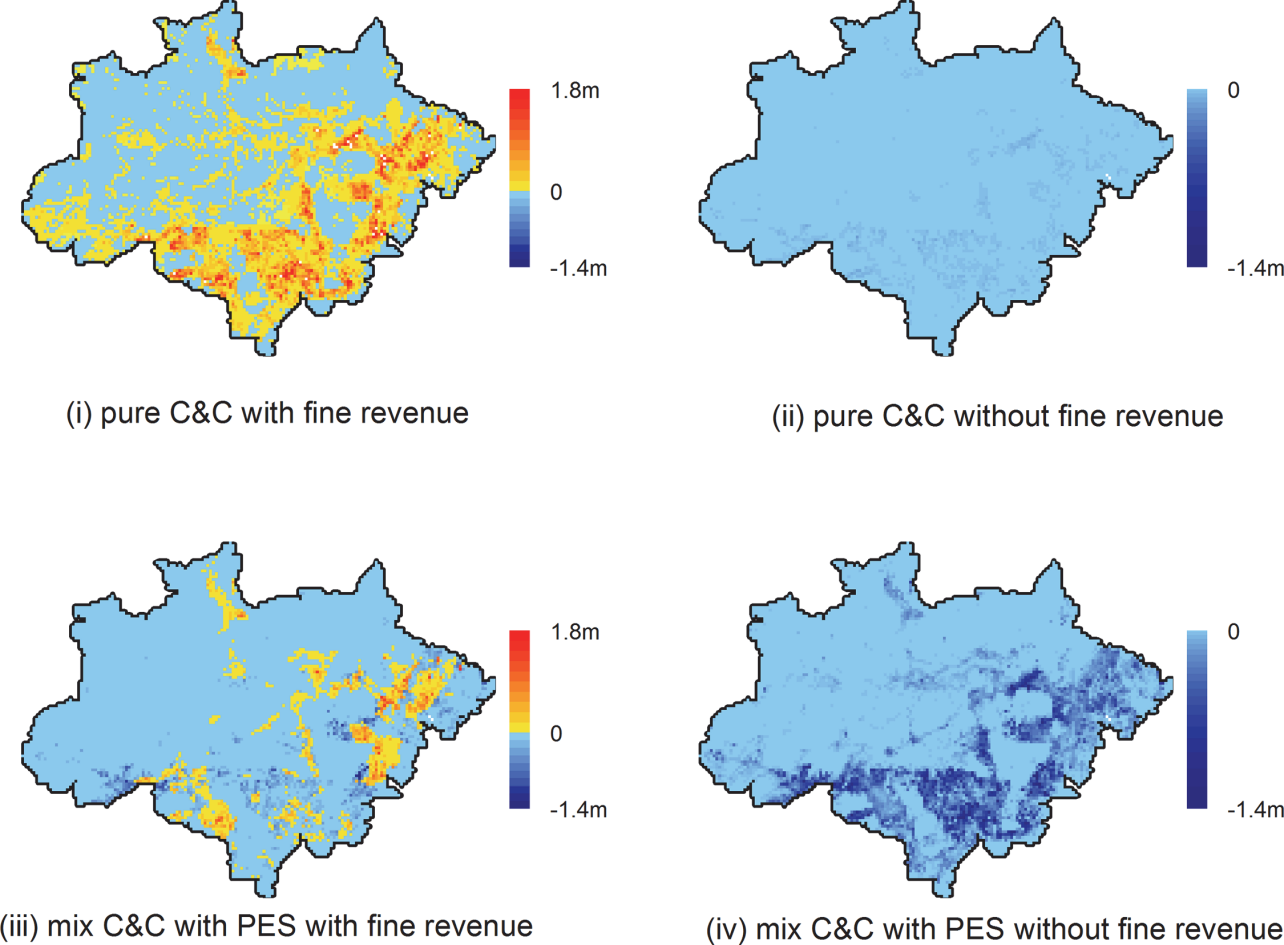
Implementation costs [BRL] per 20×20 km grid cell with (left panels) and without (right panels) fine revenues for policy mix without (upper panels) and with PES (lower panels). Parameter values: B = BRL 50 million, F = BRL/ha 5000, PES = BRL/ha 0 or 500.

Implementation costs will vary substantially across space with high enforcement costs in remote locations and high levels of deforestation. Fig. 5 depicts the spatial patterns of the EPA’s net revenue of policy implementation (*fine revenues—implementation costs*) for scenarios of C&C with and without an additional PES component (respectively upper and lower panels), as well as with and without fine collection (left and right panels). Total avoided deforestation in the pure C&C scenario is 1.4 million hectares vis-à-vis average pre-2005 deforestation—a 63% reduction. Enforcement costs for this scenario are BRL 38 million and the potential fine revenue is BRL 1.1 billion. In the C&C with PES policy mix, avoided deforestation increases to 1.5 million hectares (65% reduction), but enforcement costs including PES transfers reach BRL 1 billion, with total fine revenues slightly lower at 0.9 billion due to higher compliance levels as a result of PES. Roughly BRL 300 million of the total PES transfers are lost due to imperfect enforcement (see section 2). Nevertheless, fine collection would make this particular sticks and carrots policy mix almost cost neutral.

Without fine collection, policy implementation costs are highest in the southern and western parts of the region in what has traditionally been called the ‘arc of deforestation’ (upper right panel in Fig. 5). If the C&C strategy was complemented by PES transfers, the bulk of these transfers would then also be made to these regions, where the implementation costs of the policy mix consequently increase (lower right panel).

If the forest law was enforced primarily by collecting fines instead of other *in situ* enforcement measures (left panels), the policy mix could yield positive net financial returns for government in large parts of the region, especially in the pure C&C case. Introducing PES (lower left panel) results in fine revenues being too low to cover PES outlays in many areas. Fine revenues may cover the cost of the policy mix in places with low cost of access, where opportunity costs, and thus non-compliance levels, are high. Compliance levels in low opportunity cost areas are consequently higher, resulting in net costs of enforcement, even in the fine collection scenario.

### d) Enforcement budget and cost-effectiveness

**Fig 6:**
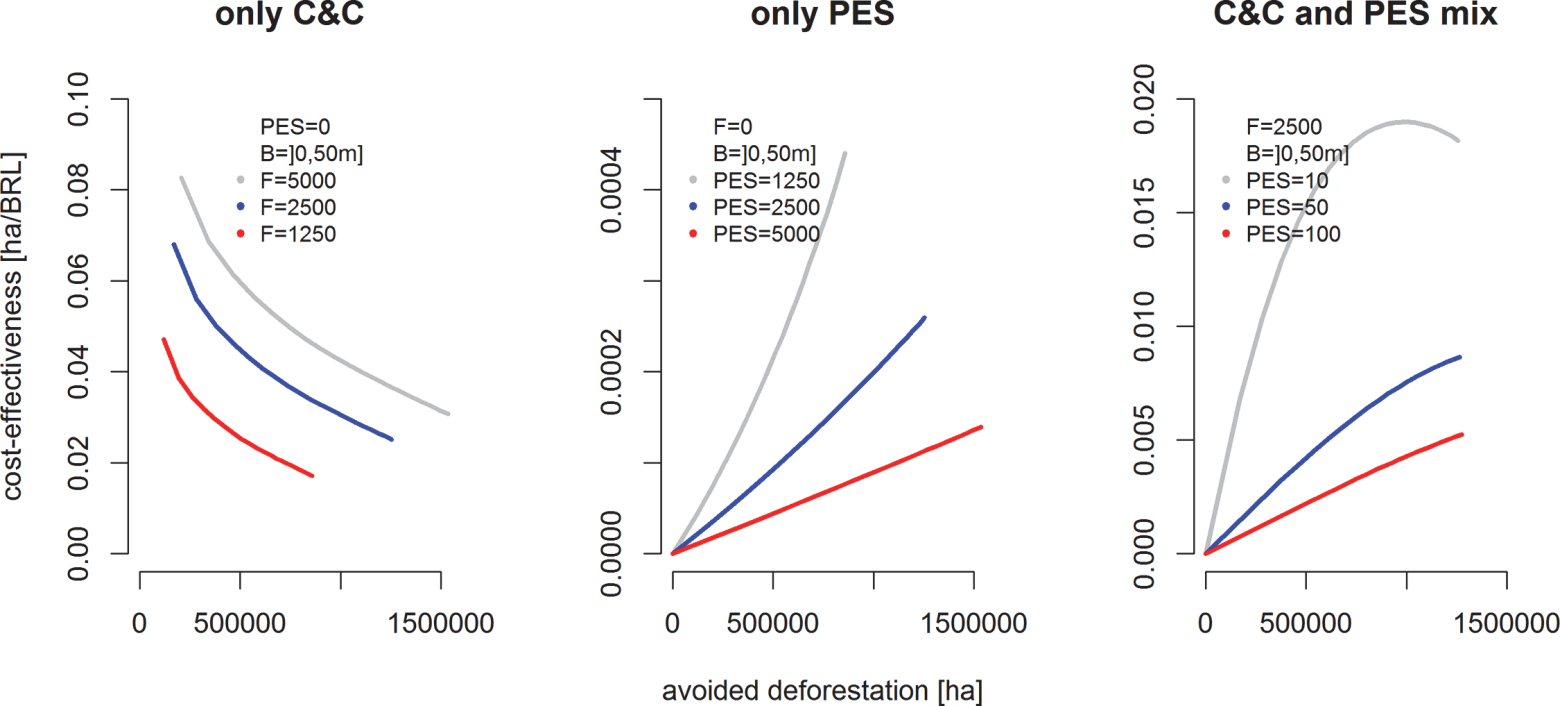
Cost-effectiveness of forest conservation at increasing conservation budgets from BRL 0 to 50 million and varying per hectare incentive/disincentive levels for three alternative policy mixes. Legend: parameter combinations used in simulations.

Increases in the enforcement budget constraint *B* (Eq. 3) will result in higher enforcement probabilities across the region. Fig. 6 shows how such changes affect the cost-effectiveness (hectares conserved per BRL invested) of alternative combinations of C&C and PES policy components.

Lines in Fig. 6 represent the cost-effectiveness of alternative avoided deforestation levels achieved through changes in the enforcement budget. In the pure C&C case with no PES (left panel) cost-effectiveness is high at low levels of avoided deforestation in particular if the budget is small. At increasing budget levels, more deforestation can be avoided, but disproportionally higher budgets are needed because additional deforestation reductions come at higher opportunity costs. All else equal, reducing the level of fines will always result in lower cost-effectiveness, but at increasing rates of reduction in cost-effectiveness (i.e., the distance between the grey and the blue line in the left panel of Fig. 6 is smaller than the distance between the blue and the red line).

In a pure PES policy scenario with no fines (center panel in Fig. 6), cost-effectiveness responds positively to increases in the enforcement budget. This is because, at low enforcement probabilities, only a small share of the PES transfer actually represents an effective incentive to avoid deforestation; a large share will *de facto* come to function as an unconditional income transfer (see section 2). At higher budget levels, cost-effectiveness therefore increases considerably, but at much lower effectiveness levels than under C&C. The cost-effectiveness pattern is similar for higher absolute levels of PES transfers, but cost-effectiveness increases at lower rates, because of increasing marginal opportunity costs.

In a combined sticks-and-carrot scenario (per-hectare fine 2500 BRL fixed, PES levels 10/50/100 BRL, respectively; right panel in Fig. 6), we observe a combination of the two individual instrumental effects especially at low levels of payments, which would appear as a more realistic short-term scenario in the Brazilian policy context. C&C and PES mutually reinforce each other resulting in higher marginal increases in cost-effectiveness at low budgets than in the pure PES case (center panel). As compliance levels increase with higher budgets, cost-effectiveness tends to level off.

### e) Income effects of a sticks-and-carrots policy mix

**Fig 7:**
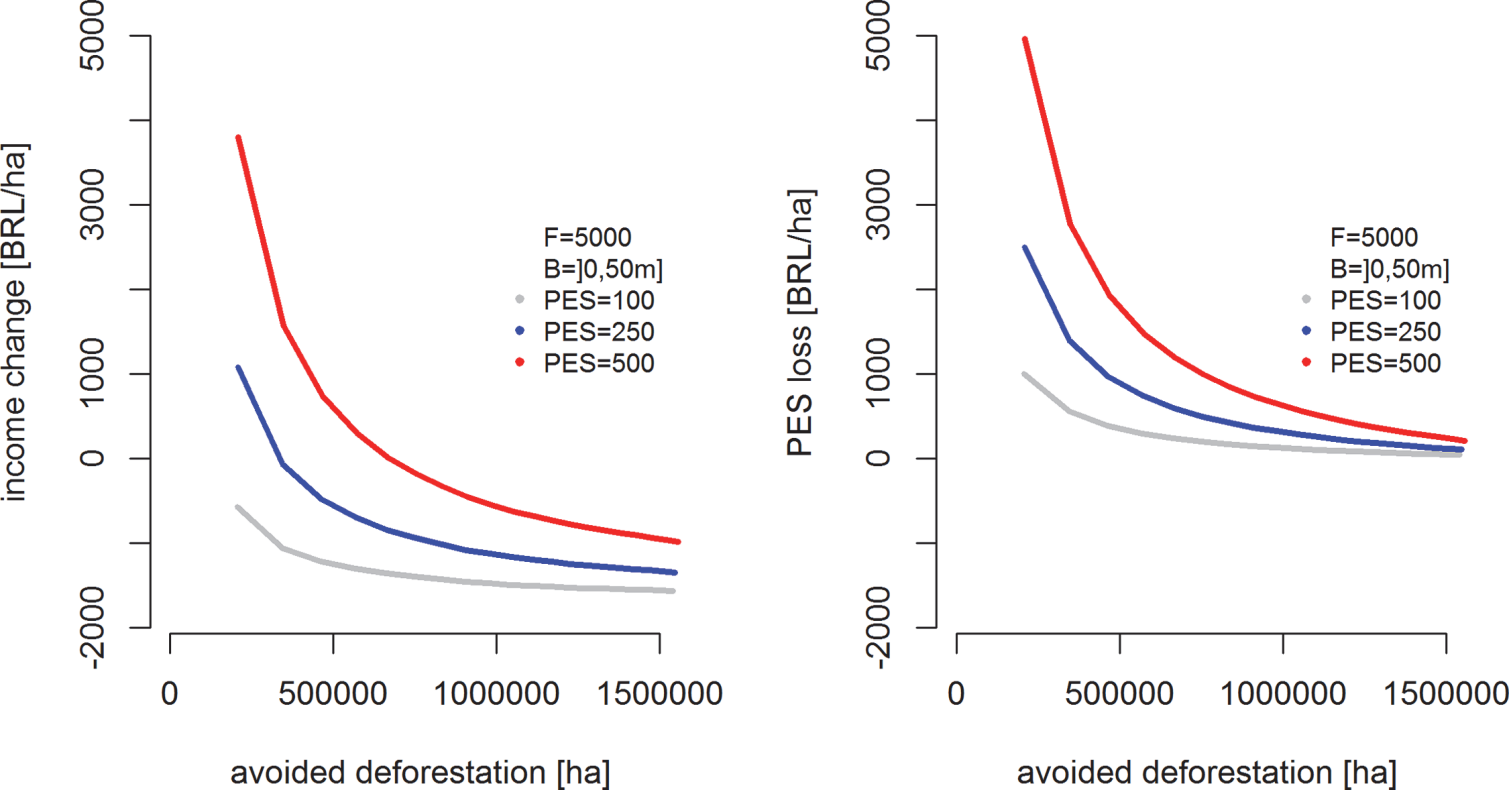
Income effect of a C&C and PES mix (left panel) and ineffective portion of PES transfers (PES loss, right panel), per hectare of avoided deforestation, with varying budget and per hectare PES values. Legend: parameter combinations used in simulations.

Introducing incentives into a C&C dominated forest conservation policy changes the income of land users according to Eq. 5. Fig. 7 (left panel) depicts average income change per hectare of avoided deforestation for varying enforcement budget levels and per-hectare conservation incentives (PES). The level of disincentives is held constant at F = 5000 BRL/ha. Increases in per hectare incentives and enforcement budget both increase avoided deforestation as suggested in Fig. 1, but in Fig. 7 the conservation effect of increasing per hectare PES is hardly visible.

Nonetheless, increases in the enforcement budget will, all else equal, always lead to lower net benefits (higher net costs) for land users. This is primarily because higher enforcement probabilities will reduce the amount of PES that is transferred unconditionally—and thus ineffectively from a conservation perspective (right panel in Fig. 7; see also Section 2). At low enforcement budgets, even relatively small increases in PES will thus result in large positive income changes, whereas the differences between the grey, blue, and red lines in the left panel of Fig. 7 tend to be smaller at the high end of the enforcement budget range. The same holds for the portion of PES payments lost as conservation-ineffective social cash transfer (PES loss, right panel).

**Fig 8:**
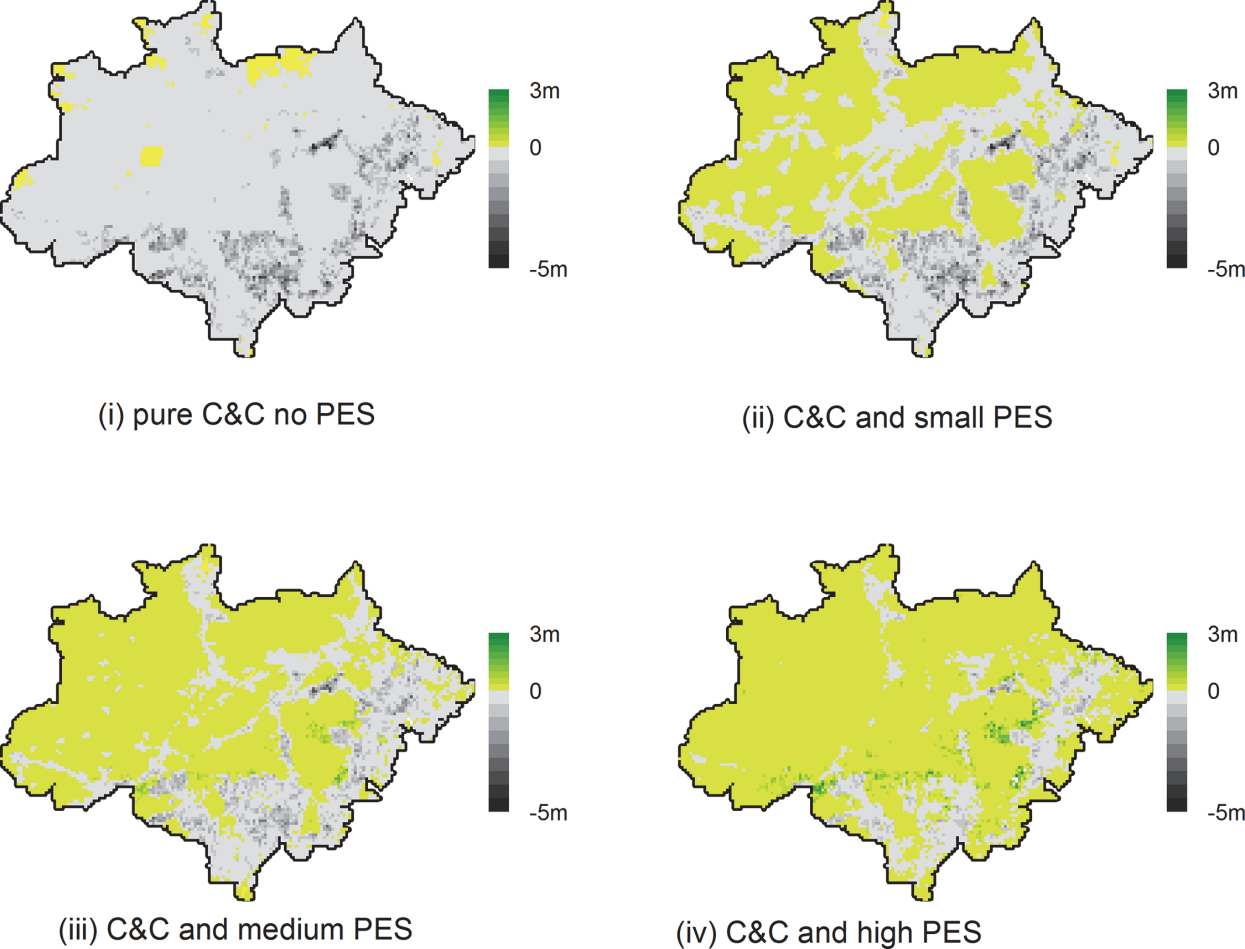
Absolute income change [BRL] per grid cell with varying levels of PES in the policy mix. Upper left: PES = BRL /ha 0; F = BRL/ha 5000, upper right: PES = BRL/ha 100; F = BRL/ha 4900, lower left: PES = BRL/ha 500; F = BRL/ha 4500, lower right: PES = BRL/ha 1000; F = BRL/ha 4000. Other parameters: B = 50m.

If policy makers accept the unconditional transfer of a share of PES in the policy mix by way of imperfect enforcement, the policy could still have important income effects. Fig. 8 depicts the spatial distribution of income changes for policy mixes with increasing PES levels. Without PES (upper left panel) most land users will face at least small negative income changes. In locations with high deforestation rates, however, total income losses per 20×20 km grid cell can reach up to BRL 5 million. No income losses will only occur in locations without baseline deforestation.

As the PES component of the policy mix increases, more and more locations exhibit zero or positive absolute income changes, especially in remote deforestation sites, where enforcement probabilities are low. Where conservation opportunity costs are high, compliance levels will be low and so will be the benefits of PES. Some locations will thus exhibit high income losses even at relatively high PES levels (lower right panel in Fig. 8). High income gains will consequently accrue to compliant land users with low opportunity costs.

Depending on the distribution of farms and farm sizes (not analyzed here) as well as conservation opportunity costs across the intervention area, PES also might boost the equity outcome of an otherwise C&C dominated forest conservation policy. Remotely located land users with low opportunity costs (and thus probably low historical deforestation records) will be disproportionally rewarded through PES as they tend to face low enforcement probabilities. Land users with high opportunity costs, on the other hand, would be hit disproportionally hard by the C&C disincentive, but still be partially compensated through PES.

## Discussion

Brazil is currently at the verge of introducing more forest conservation incentives, to complement pre-existing and, in the past decade, markedly more efficiently functioning command-and-control systems. We have thus developed an economically motivated probabilistic spatial simulation model to assess the likely outcomes of various anti-deforestation policy mixes involving C&C and PES components: Amazon land users are still subject to fines when they illegally deforest (and get caught), but the bar is raised for them in the sense that they can also receive PES (functioning basically as compliance subsidies), which they equally risk losing when being caught in illegal deforestation. We scrutinize the impacts of various combinations of positive and negative incentives, in terms of both environmental cost-effectiveness and land user incomes. The model extends previous work [27] in two ways: (1) we add the combined effects of ‘carrots’ on top of ‘sticks’, and (2) we model conservation decisions as a continuous function of conservation opportunity costs.

The advantages of our modeling approach (relatively high spatial resolution and economically motivated land use and enforcement decisions) naturally involve some tradeoffs. Our measure of conservation opportunity costs, for example, allows us to study the effects of commodity price changes only in aggregate terms and interactions with the rest of the economy (forward linkages, multiplier effects) are ignored. These limitations could in principle be overcome by integrating our approach with existing rule-based agent or partial and general equilibrium models.

Moreover, our approach to model law enforcement differs from the notion of optimal enforcement in the enforcement literature [31] in several aspects. Most notably, we do not consider the law enforcement strategy of the financial and administratively autonomous Brazilian EPA to be motivated by social welfare criteria (see also, [28]). Instead we model the EPA as an environmental principal with an exogenous budget, whose resource allocation decisions are not affected by the economic opportunity costs of its actions. This approach reflects a short-run perspective on the forest law enforcement strategy in the Brazilian Amazon, including related institutional limitations [27]. In the long-run, government budgets for environmental control and enforcement are bound to be endogenously determined by various economic and political factors, including interest groups composed of those affected by enforcement. Our approach is not suited to predict this potential long-term social welfare-maximizing equilibrium. It is rather designed as an ex-ante impact assessment tool to illustrate the range of alternative environmental policy outcomes, in terms of cost-effectiveness and land user income, at much higher levels of spatial detail than standard computable equilibrium models.

Our analysis puts C&C policies clearly in the driver’s seat of achieving environmental cost efficiency, while PES policies become more of a complementary tool that reinforces effectiveness and shares the costs of compliance more equally, thus making avoided deforestation politically more palatable. But, as indicated above, the model of PES policies we have used for this initial policy-mix analysis is rudimentary. We assumed that all land users could be subjected simultaneously to both C&C and PES ‘treatments’; yet we know that PES is a demanding tool in terms of institutional preconditions, especially in terms of land stewards’ ability to exclude third party access so as to safeguard environmental service provision [30]. In an earlier analysis, we found that in up to two thirds of the Brazilian Amazon, PES will be difficult to implement due to insecure or unclear tenure [6]. While that percentage may since have been reduced somewhat by Brazil’s extensive efforts to regularize Amazon land tenure, a large share of threatened forestland does still not present the necessary preconditions for PES. In addition, to many observers it may not be politically acceptable to use PES as a generalized compliance subsidy, i.e. of paying people to abide to the law at a regional scale. Hence, PES may instead end up being offered to only a politically filtered sub-spectrum of land users, including because of its potentially heavy costs. Finally, we have assumed rather optimistically that we can predict precisely which areas would be at risk of deforestation, so that all PES contracts would become fully additional in avoiding deforestation. This ignores non-trivial challenges in spatially predicting deforestation, including in avoiding leakage and the adverse selection bias that is typical for a voluntary instrument like PES [32, 33].

Explicitly modelling a more sophisticated PES design in our policy mix simulation is unlikely to affect the overall tradeoff dynamics identified above, but it may affect its dimensions. For example, modelling additional PES transaction cost components, such as contract negotiation and other administrative costs, would further reduce the cost-effectiveness of a C&C with PES policy mix. On the other hand, PES could be modelled as a more cost-effective policy component by accounting for critical design factors, such as (1) *Spatial targeting of contracts* (excluding areas with low deforestation pressure and environmental service values) or (2) *Differentiated payments* (according to varying levels of opportunity costs), and (3) *equity effects* [34–36]. Modelling these dimensions of environmental policy instruments at sub-district spatial scales requires more detailed information about the spatial distribution of property rights and welfare levels that is only slowly emerging in our study area [37, 38]. On the other hand, having to pay in reality also for some non-additional (i.e. not immediately threatened) forestlands would make PES more expensive vis-à-vis C&C, thus exacerbating the cost implications we have analysed above.

### Conclusions and policy implications

To design an adequate policy mix for the management of land-use and land-cover change in tropical forest landscapes, policy makers have to simultaneously maneuver several policy design parameters. Growing interest exists to add more positive incentives to the anti-deforestation policy mix in the Brazilian Amazon. Hence, we have examined some ‘carrot’ (PES) and ‘stick’ (C&C) policy mixes with respect to three parameters: (1) enforcement agency budgets, (2) disincentives vis-à-vis incompliant land users, and (3) incentives provided to compliant land users. Our analysis confirms that policy makers will face hard tradeoffs in aligning income-enhancing incentives (PES) with income-reducing but cost-effective disincentives (C&C), in order to make conservation socially acceptable. We find that a purely C&C dominated forest conservation policy could achieve conservation gains of a size equivalent to the drop in Amazon deforestation levels between 2004–2012 at enforcement costs of only BRL 0.03 per hectare of forest conserved. But, the opportunity costs borne by land users in this scenario exceed BRL 2 billion annually.

Mixing incentives and disincentives instead, such that land users were, at least on average, compensated for avoiding the same level deforestation would reduce cost-effectiveness by over 98%. Our simulations suggest that monitoring and enforcement capacity will play a crucial role in determining what share of the incentives will actually result in additional conservation. If the Brazilian government offered Amazon land users PES values in the range of BRL 100–500 per ha, our analyses shows that losses due to imperfect enforcement may lie in the range of BRL 200–5000 per hectare of avoided deforestation. The actual amount depends on the level of C&C disincentives and the budget of the enforcement agency. The reason is that imperfect enforcement renders a share of the PES payment ineffective from a conservation perspective.

In favorable local intervention contexts, nonetheless, PES appears as a complementary instrument to make conservation policies more equitable and less of a burden to land users. Like the Brazilian Amazon region, many tropical forest frontiers are characterized by a high concentration of land ownership and income with large landholders causing the lion’s share of deforestation. Conservation incentives can here only be cost-effective if large landholders obtain a major share of conditional payments. Carrots without sticks are thus likely to be met with opposition by landless peasants or traditional forest stewards who cannot easily demonstrate additionality on the basis of land tenure or historical deforestation records. Conversely, C&C dominated strategies may mobilize powerful agricultural lobbies to weaken conservation standards, as we have also seen in Brazil. Introducing incentives in locations where high levels of monitoring and enforcement can be assured may be a promising strategy to balance the costs and benefits of conservation across multiple stakeholder groups.

Finally, lessons can be drawn with respect to more sophisticated conservation policy instruments, such as tradable development rights. According to Brazil’s New Forest Code, land users that exceed the legal share of deforested land on their properties may be allowed to trade development rights with land users who dispose of excess forestland, although trading rules have not yet been specified [37]. Tradable development rights essentially convert regulations into conservation incentives and disincentives in spatially separated locations. High-deforestation locations with large land demand can pay a “tax” to buy development rights from low-deforestation locations a conservation “subsidy”. Yet, both sellers and buyers of development rights must be monitored and punished if they fail to comply with the reallocation of development rights after trade. In the absence of effective monitoring and enforcement on the high-deforestation demand side, demand will fail to materialize, and the trade system will be ineffective. If enforcement is weak on the supply side, trade can take place, but excess deforestation may not be offset by conservation in supplying areas. If enforcement strategies follow the empirically motivated logic of our simulation model (see S1 Appendix), our scenario analyses point to inefficiencies due to imperfect supply side enforcement. Implementing tradable development rights in the Brazilian Amazon region would thus probably require an alignment of enforcement strategies with the expected regional patterns of trade in development rights.

Conservation incentives are now being endorsed as a key elements of a “new model of rural development” for the Brazilian Amazon ([7], p. 1123). Effectively implementing them in concert with existing regulations is technically feasible, and probably also socially desirable. Their cost-effectiveness and income effects, however, will largely depend on decision-makers’ capacity to design a balanced conservation policy mix, and will not exonerate them from maintaining a sophisticated command-and-control system of compliance monitoring and sanctions.

## Supporting Information

S1 Appendix The enforcement strategy of the Brazilian Environmental Protection Agency IBAMA. (DOC)

S2 Appendix Opportunity cost estimation. (DOC)

S1 Data Supporting data. (ZIP)
