# Supplementary material for: Mixing Carrots and Sticks to Conserve Forests in the Brazilian Amazon: A Spatial Probabilistic Modeling Approach
Source: PLoS One. 2015 Feb 4;10(2):e0116846. doi: 10.1371/journal.pone.0116846 (PMC4317180; doi:10.1371/journal.pone.0116846)
Supplement: S1 Appendix — (DOC) [file pone.0116846.s001.doc]

**Appendix S1: The enforcement strategy of the Brazilian Environmental Protection Agency IBAMA**

In section 2 above, we hypothesize that the Brazilian EPA seeks to maximize the area of inspected deforestation patches subject to a budget constraint. Inspection costs should thus be negatively influencing the probability of inspections, whereas the extent of deforestation should positively correlate with inspection probability. To empirically verify these expectations we rely on 4169 spatial point coordinates of field inspections in the year 2009. We estimate a logistic regression model of the form:

$\log\frac{\Pr\left(x\right)}{1-\Pr\left(x\right)}=\beta_{0}+\beta_{1}Dist+\beta_{2}d+\beta_{3}NI+\varepsilon$ Eq. (A1)

*Dist* is the travel distance to (see Fig. 1) and *d* is the total remotely sensed deforestation in each grid cell in the year 2009. Our dependent variable is 1 if one or more inspections were made in a grid cell and 0 otherwise. *NI* is an indicator for inspections in neighboring grid cells to account for spatial autocorrelation. Figure A1 shows the results from estimating Eq. A1 suggesting that our approach to simulate IBAMA’s enforcement strategy correctly reflects the underlying tradeoff between inspection costs and objectives.


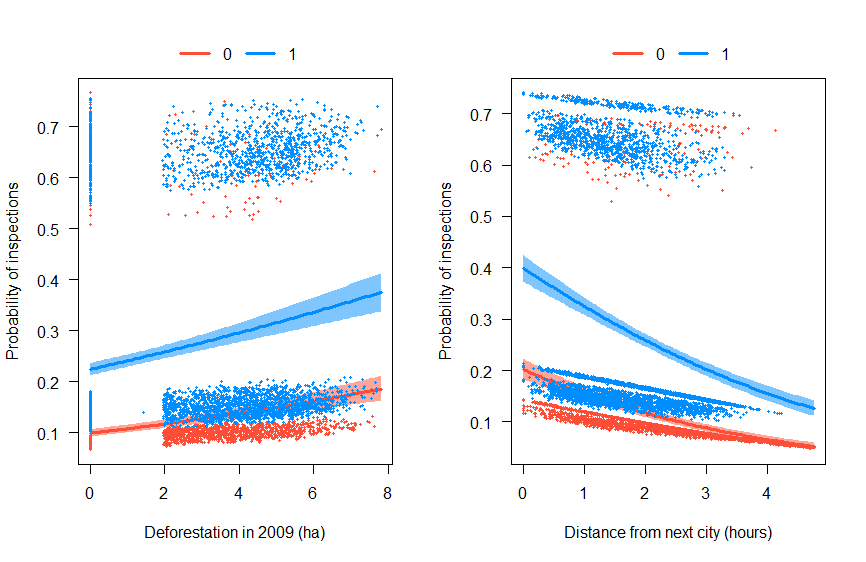
**Figure A1: Probability of inspections as a function of deforestation (left) and travel distance to next district center. Red and blue lines distinguish between isolated inspections (no neighboring cells inspected) and clustered inspections (neighboring cells inspected), respectively.**
