# Supplementary material for: Mixing Carrots and Sticks to Conserve Forests in the Brazilian Amazon: A Spatial Probabilistic Modeling Approach
Source: PLoS One. 2015 Feb 4;10(2):e0116846. doi: 10.1371/journal.pone.0116846 (PMC4317180; doi:10.1371/journal.pone.0116846)
Supplement: S2 Appendix — (DOC) [file pone.0116846.s002.doc]

**Appendix S2: Opportunity cost estimation**

The potential gain one expects from the conversion of a tree cover into a non-forest use is what dictates the deforestation process: a given area in a given step of time will be deforested if and only if the revenue of deforestation is higher than its cost. Although straightforward, this statement –derived from the premise of profit maximizing agents – has strong implications on how to model the opportunity costs of deforestation (henceforth opportunity costs). First, their spatial heterogeneity should follow, at least in some extent, the spatial heterogeneity of deforestation. Second, opportunity costs over an area and in a given step of time are strictly positive or negative only if deforestation rates are 100% and 0%, respectively. In other words, assuming that labor and capital inputs for forest clearing are mobile, the proportion of the area deforested equals the proportion of the area with positive opportunity costs. Third, opportunity costs, in their most general view, are as dynamic as the deforestation itself. Indeed, if opportunity costs are constant over time, deforestation will happen all at once and stop in the next time step, because encroaching on the remaining forested areas would generate negative revenues. Clearly, this only holds if all revenues and costs associated with deforestation are taken into account, including existing bio-physical or policy related constraints. This assumption is the basis for our strategy for the empirical estimation of the distribution of opportunity costs within grid cells. It establishes a theoretically consistent relationship between observed deforestation rates at the sub-district level with district-level estimates of average deforestation opportunity cost.

Without loss of generality, we define the spatial scale for the estimation of opportunity costs distribution as 20x20km grid cells. Let us assume that opportunity costs distribution within a grid cell can be approached by a normal distribution:

$R_{g,m,t}\sim N\left(\mu_{g,m,t},\sigma_{g,m,t}\right)$ (1)

Where *R_g,m,t_* is the vector of opportunity costs (or net revenues from deforestation), at grid cell *g*, municipality *m* and time *t*; and *μ_g,m,t_* and *σ_g,m,t_* are the parameters of the normal distribution with their respective grid cell, municipality and time indices. The same standards for subscripts are adopted throughout the description of the methodology with no explicit explanation.

The deforestation rate (*DR*) in time *t* is defined by the deforested area (*DA*) in time *t* divided by the total forested area (*FA*) in time *t-1*:

$DR_{g,m,t}=\frac{DA_{g,m,t}}{FA_{g,m,t-1}}$ (2)

As deforestation only happens in areas where *R*>0 follows that:

$DR_{g,m,t}=\Pr\left(R_{g,m,t}>0\right)$ (3)

In other words, the deforestation rate in a grid cell corresponds to the probability of *R_g,m,t_* of being positive. Consequently, for each deforestation rate there is a family of normal distributions characterized by a given μ/σ rat

$1-\Phi\left(\frac{\mu_{g,m,t}}{\sigma_{g,m,t}}\right)=\Pr\left(R_{g,m,t}>0\right)$ (4)

$\frac{\mu_{g,m,t}}{\sigma_{g,m,t}}=\Phi^{-1}\left(1-\Pr\left(R_{g,m,t}>0\right)\right)$ (5)

Where
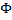
 and
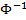
 have been respectively defined as the cumulative density function of a normal distribution and its inverse. And by replacing (3) into (5), the family of normal distributions can be derived from the deforestation rate:

$\frac{\mu_{g,m,t}}{\sigma_{g,m,t}}=\Phi^{-1}\left(1-DR_{g,m,t}\right)$ (6)

Now, given that measured revenues from deforestation come from actually deforested areas, the average opportunity cost
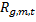
 in a given grid cell is given by the expected value of the positive part of opportunity cost distribution:

$\overline{R}_{g,m,t}=E\left(R_{g,m,t}|R_{g,m,t}>0\right)$ (7)

At this point, it is worth noting that
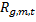
 is fully determined by *μ_g,m,t_* and *σ_g,m,t_*. Although their ratio is known by inputting deforestation rates into (6), additional information is needed for the estimation of their levels. Given that actual information on opportunity costs (or deforestation revenues) is aggregated at the municipality level, we first assume homogeneity of variance among grid cells of the same municipality as a way to tie the families of normal distribution within a municipality:

(8)

Where *G^m^* is the set of grid cells within the municipality *m*. Finally, we define the average opportunity cost aggregated at the municipality level as the average of the opportunity costs of the grid cells within the municipality weighted by the deforested area in each grid cell:

$\overline{R}_{m,t}=\sum_{G^{m}} \frac{\overline{R}_{g,m,t}*DA_{g,m,t}}{\sum_{G^{m}} DA_{g,m,t}}$ (9)

Follows that for each municipality there is only one *σ_g,m_* that generates a set of *μ_g,m,t_* (that varies among cells through (6)) that fulfils (9) given the actual estimates of opportunity costs.
